# Mosquito Transcriptome Profiles and Filarial Worm Susceptibility in *Armigeres subalbatus*

**DOI:** 10.1371/journal.pntd.0000666

**Published:** 2010-04-20

**Authors:** Matthew T. Aliota, Jeremy F. Fuchs, Thomas A. Rocheleau, Amanda K. Clark, Julián F. Hillyer, Cheng-Chen Chen, Bruce M. Christensen

**Affiliations:** 1 Department of Pathobiological Sciences, University of Wisconsin-Madison, Madison, Wisconsin, United States of America; 2 Department of Biological Sciences and Institute for Global Health, Vanderbilt University, Nashville, Tennessee, United States of America; 3 Department of Microbiology and Immunology, National Yang-Ming University, Taipei, Taiwan Authority; National Institute of Allergy and Infectious Diseases, United States of America

## Abstract

**Background:**

*Armigeres subalbatus* is a natural vector of the filarial worm *Brugia pahangi*, but it kills *Brugia malayi* microfilariae by melanotic encapsulation. Because *B. malayi* and *B. pahangi* are morphologically and biologically similar, comparing *Ar. subalbatus-B. pahangi* susceptibility and *Ar. subalbatus-B. malayi* refractoriness could provide significant insight into recognition mechanisms required to mount an effective anti-filarial worm immune response in the mosquito, as well as provide considerable detail into the molecular components involved in vector competence. Previously, we assessed the transcriptional response of *Ar. subalbatus* to *B. malayi*, and now we report transcriptome profiling studies of *Ar. subalbatus* in relation to filarial worm infection to provide information on the molecular components involved in *B. pahangi* susceptibility.

**Methodology/Principal Findings:**

Utilizing microarrays, comparisons were made between mosquitoes exposed to *B. pahangi*, *B. malayi*, and uninfected bloodmeals. The time course chosen facilitated an examination of key events in the development of the parasite, beginning with the very start of filarial worm infection and spanning to well after parasites had developed to the infective stage in the mosquito. At 1, 3, 6, 12, 24 h post infection and 2–3, 5–6, 8–9, and 13–14 days post challenge there were 31, 75, 113, 76, 54, 5, 3, 13, and 2 detectable transcripts, respectively, with significant differences in transcript abundance (increase or decrease) as a result of parasite development.

**Conclusions/Significance:**

Herein, we demonstrate that filarial worm susceptibility in a laboratory strain of the natural vector *Ar. subalbatus* involves many factors of both known and unknown function that most likely are associated with filarial worm penetration through the midgut, invasion into thoracic muscle cells, and maintenance of homeostasis in the hemolymph environment. The data show that there are distinct and separate transcriptional patterns associated with filarial worm susceptibility as compared to refractoriness, and that an infection response in *Ar. subalbatus* can differ significantly from that observed in *Ae. aegypti*, a common laboratory model.

## Author Summary

In general, organisms can use two different strategies when confronted with pathogens, tolerance and/or resistance. Resistance reduces the fitness of the invading pathogen, whereas tolerance reduces the damage caused by the pathogen to the host. Mosquitoes that transmit the parasites that cause human lymphatic filariasis generally are tolerant to the parasite, whereas those that do not transmit the parasite are resistant. We examined the effects of filarial worm tolerance and resistance on *Armigeres subalbatus* by analyzing changes in mosquito gene expression at key stages of parasite development and destruction. Because the gene expression data showed few mosquito transcriptional changes associated with parasite development, we morphologically examined mosquito flight muscle to see if we could identify damage associated with parasite infection. The research described in this manuscript provides a better understanding of the molecular components involved in compatible and incompatible relationships between mosquitoes and the filarial worm parasites that they transmit; and it will provide new insights into the complex biology of vector competence and the origins of host defense, and possibly lead to the functional characterization of previously unknown gene products involved in vector competence.

## Introduction

Human lymphatic filariasis (LF) is caused by several species of mosquito-borne filarial nematodes, including *Brugia malayi*, *Brugia timori*, and *Wuchereria bancrofti*. Lymphatic filariasis is not a disease that causes mortality but it is by no means trivial. It is estimated that 120 million people in the world have LF, with ∼1.1 billion at risk of becoming infected. Although LF is rarely fatal, severe morbidity (including adverse economic and psychosexual effects) involves disfigurement of the limbs and male genitalia (elephantiasis and hydrocele, respectively) [1]–[3]. Mosquitoes belonging to a number of different genera, including *Culex*, *Aedes*, *Mansonia*, *Anopheles*, and *Armigeres*, can serve as competent vectors. Following ingestion of an infective bloodmeal, microfilariae (mf) penetrate the mosquito midgut epithelium and migrate to the thoracic musculature. Migration to the head region occurs after a series of larval molts to the infective third stage (L3), which occurs in thoracic muscle cells. The L3 is then passed to a vertebrate host when the infected mosquito takes a bloodmeal. In these mosquito-parasite systems, the coevolutionary history of parasite and vector in one geographic region can differ from vector-parasite relationships in another area [4].

The ability of vector mosquitoes to ingest mf of filarial worm parasites and to support their development after ingestion is an important determinant of filariasis transmission. For a plethora of reasons, often times the number of L3s developing from ingested mf is not constant [5]. Resistance to mf in the mosquito mainly involves melanotic encapsulation (e.g., *Ar. subalbatus* and *B. malayi*, *Aedes trivitattus* and *Dirofilaria immitis*, *Anopheles quadrimaculatus* and *B. pahangi*), but permissiveness of the midgut for parasite penetration, and physiological suitability of the thoracic muscle cells also may be important determinants of vector competence [6]–[9]. When melanization occurs, mf are rapidly melanized in the hemocoel once they have penetrated the midgut. As soon as 10 minutes following a bloodmeal, melanin deposition is evident on the mf cuticle. At 12–16 hours post feeding, melanization is well underway and pathological effects on the mf are evident. At 24 to 48 hours post feeding mf begin to die, and by 72 hours post feeding, the response is all but complete [7], [8].

*Armigeres subalbatus* is a natural vector of the filarial worm *Brugia pahangi*, but it kills *B. malayi* mf by melanotic encapsulation [6]. Because *B. malayi* and *B. pahangi* are morphologically and biologically similar, this mosquito-parasite system serves as a valuable model for studying resistance mechanisms in mosquito vectors [10]. Previously, we assessed the transcriptional response of *Ar. subalbatus* to *B. malayi*, which revealed the possible involvement of a number of unknown and conserved unknown gene products, cytoskeletal and structural components, and stress and immune responsive factors in the mosquito's anti-filarial worm response. The data showed that the anti-filarial worm immune response by *Ar. subalbatus* is a highly complex, tissue-specific process involving varied effector responses working in concert with blood cell-mediated melanization [11]. Therefore, comparing *Ar. subalbatus-B. pahangi* susceptibility and *Ar. subalbatus-B. malayi* refractoriness could provide significant insight into recognition mechanisms required to mount an effective anti-filarial worm immune response in the mosquito, as well as provide considerable detail into the molecular components involved in vector competence.

Accordingly, we initiated transcriptome profiling studies of *Ar. subalbatus* in relation to filarial worm infection to provide information on the molecular components involved in *B. pahangi* susceptibility for comparison with our earlier studies on *B. malayi* refractoriness [11]. In addition, these studies also provide information on the infection response of a natural vector, i.e., the overall transcriptional and physiological change that occurs in the mosquito as a result of parasite infection, for comparison with our previous studies that employed a highly susceptible laboratory model, *Aedes aegypti* [12]. The time course chosen facilitated an examination of key events in the development of the parasite, beginning with the very start of filarial worm infection and spanning to well after infective-stage parasites had completed development in the mosquito. The data presented herein provide us with a cadre of information to design wet lab experiments and select candidates for further study to more fully dissect the nature of the anti-filarial worm immune response in this mosquito-parasite system. And with no genome sequence available for *Ar. subalbatus*, these data sets, in conjunction with data generated from our previous work (see [11]), represent the most complete set of transcriptomic information available to date for this mosquito species, which should be of interest to numerous laboratories investigating vector biology and innate immunity in general.

## Methods

### Mosquito maintenance

*Ar. subalbatus* used in this study were maintained at the University of Wisconsin-Madison as previously described [7]. *Ar. subalbatus* supports the development of *B. pahangi* mf to L3 [6], [13]. Three- to four-day-old female mosquitoes were used for bloodfeeding and sucrose starved for 14 to 16 hours prior to this event.

### Exposure to infective bloodmeal

All animals and animal facilities are under the control of the School of Veterinary Medicine with oversight from the University of Wisconsin Research Animal Resource Center, and their use in experimentation was approved by the University of Wisconsin Animal Care and Use Committee. Mosquitoes were exposed to *B. pahangi* and *B. malayi* (originally obtained from the University of Georgia NIH/NIAD Filariasis Research Reagent Repository Center) by feeding on ketamine/xylazine anesthetized gerbils, *Meriones unguiculatus*. The same animals were used for all three biological replicates. Microfilaremias were determined, using blood from orbital punctures, immediately before each feeding and ranged from 15–60 mf/20 µl blood. Control mosquitoes were exposed to anesthetized, uninfected gerbils. Mosquitoes that fed to repletion were separated into cartons and maintained on 0.3 M sucrose in an environmental chamber at 26.5°±1°C, 75±10% RH, and with a 16 hr photoperiod with a 90 minute crepuscular period.

### Mosquito collection and verification of parasite infection

Nine sample groups were created from thirteen timepoints to study mosquito transcriptome changes in response to *B. pahangi* development. In each sample group, comparisons were made between mosquitoes exposed to an infective bloodmeal containing *B. pahangi* mf and those exposed to a bloodmeal without parasites. In addition, a separate set of microarray analyses directly compared transcriptome profiles between mosquitoes in which filarial worms develop to infective stage larvae (*B. pahangi*) and mosquitoes in which an anti-filarial worm immune response had been initiated (*B. malayi*). This direct comparison contained groups of mosquitoes exposed to an infective bloodmeal containing *B. pahangi* mf or exposed to a bloodmeal containing *B. malayi* mf. Sample collection followed the same time course chosen for the DNA microarray experiments investigating *B. malayi* refractoriness previously published [11], but included three new biological replicates of mosquitoes exposed to *B. malayi*, done concurrently with the *B. pahangi* exposures. This direct comparison was advantageous because indirect comparisons of separate experimental data sets that employ a control that is assumed to be the same treatment for each could yield false information. For example, indirect comparisons made between DNA microarrays that compared *B. pahangi* vs. blood in this study compared to *B. malayi* vs. blood in [11] could be problematic, because the variation in the separate control groups are reflected in these comparisons, and because of the variation inherent in indirect comparisons [14].

The sample groups were defined by the time post ingestion (PI) of the bloodmeal and represent significantly different stages of parasite development. Twenty mosquitoes, exposed to either *B. pahangi* or uninfected blood meals, were collected at 1, 3, 6, 12, and 24 h PI and ten mosquitoes at 2, 3, 5, 6, 8, 9, 13 and 14 d PI. Twenty mosquitoes, exposed to a bloodmeal containing *B. malayi* mf, were collected at 1, 6, 12, and 24 h PI and ten mosquitoes at 2 and 3 d PI. Mosquitoes were pooled (2–5 mosquitoes/tube), RNA was immediately extracted, and then stored at −80°C until cDNA synthesis.

At each time point, an additional 5 mosquitoes were dissected, and the head, thorax, midgut and abdomen were examined microscopically to verify filarial worm infection and to determine the stage of parasite development. Briefly, at 1, 3, 6, 12 and 24 h PI mf penetrate the mosquito midgut, migrate through the hemocoel, and penetrate thoracic muscle cells. Group 6 was collected at 2–3 d PI, a time when mf differentiate into intracellular first-stage larvae (L1). At 5–6 d PI, *B. pahangi* complete the molt to second-stage larvae (L2) and actively feed on mosquito muscle tissue (Group 7). In Group 8, collected at 8–9 d PI, parasite development is complete with a second molt to the L3, which breaks out of the thoracic muscles and migrates to the head and proboscis. The final sample collection (Group 9) was made at 13–14 d PI, when the majority of parasites have completed their migration to the head and proboscis [12], [13], [15].

### Microarray design

Please note that the terminology used to define the components of the DNA microarray are derived from the original DNA microarray paper [16]; therefore, the target is that which is tethered to the DNA microarray substrate and the probe is the labeled material in solution that hybridizes to the target. Microarrays used in this study were designed as previously described [11], [17] and printed at the University of Wisconsin Gene Expression Center using a Genomic Solutions GeneMachines Omnigrid arrayer and SMP3 pins from TeleChem International following established printing protocols.

### cDNA synthesis and purification of amino allyl-modified cDNA

RNA was collected for all microarray analyses from whole female *Ar. subalbatus* and processed as described previously [11]. Briefly, three biological replicates, each with two technical replicates, done as dye swapped pairs (Cy5 experimental vs. Cy3 control), were performed for each experimental set in an effort to eliminate dye bias [18], [19]. Each time point for each biological replicate consisted of twenty pooled mosquitoes, and each biological replicate consisted of mosquitoes from distinct generations to take into account stochastic variations. RNA integrity was verified via gel electrophoresis or via Bioanalyzer (Agilent, Santa Clara, CA) and only quality intact RNA was used for cDNA synthesis. cDNA synthesis was done according to the Chipshot™ Indirect Labeling System with modification (use of an anchored oligo(dT) primer) (TM261, Promega). Priming with anchored oligo(dT) directed the start of synthesis from the 5′ end of the poly-A tail. Twenty µg of total RNA were used as a template for the synthesis of amino allyl-modified cDNA.

### Coupling and purification of CyDye labeled cDNA

Purified cDNA from each synthesis reaction was coupled to Cy3 or Cy5 according to manufacturers' instructions. The CyDye (GE Healthcare) probes were purified using the ChipShot™ Membrane Clean-Up system (TM261, Promega). Purified, dye-coupled cDNA was measured at 260 nm (Cy5 @ 650 nm and Cy3 @ 550 nm) to calculate yield. Probes (10 pmol/dye/slide) were dried down using a speedvac, resuspended at room temperature in 45 µl Pronto!™ hybridization buffer, incubated at 95°C for 5 minutes, and applied to the DNA microarrays. DNA microarrays were hybridized overnight at 42°C. Hybridized DNA microarrays were processed using the Pronto! microarray hybridization kit (Corning) according to manufacturer's specifications.

### Microarray analysis

The microarray data were prepared according to “minimum information about a microarray experiment” (MIAME) recommendations, deposited in the Gene Expression Omnibus (GEO) database, and can be accessed via the web (accession number GSE20205). All transcript and EST data for this project are publicly accessible in ASAP (A Systematic Annotation Package for community analysis of genomes) [20] as the complete collapsed set (ARALL v2) or through NCBI's GenBank database (accession numbers EU204979-EU212998) via the web. Microarray scanning and analysis was conducted as described previously [11]. Briefly, signal intensities were normalized using GeneSpring GX 7.3.1 software. All slides were normalized using a global linear regression (Lowess) curve fit to the log-intensity vs. log-ratio plot, and 20% of the data were used to calculate the Lowess fit at each point. All data were averaged for replicate spots upon a slide, and then further averaged across slides. Minimum and maximum values were recorded and t-test p-values generated for all replicate sets. Genes showing differential expression over controls were isolated using volcano plots at a 95% confidence interval over 2-fold values. Tests were parametric, but all variances were considered equal.

### Quantitative PCR

Transcript levels of seven selected genes were measured using SYBR dye technology and quantitative polymerase chain reaction (qPCR) to validate microarray results as previously described [11]. Group 1 included three transcripts at 1 hour post challenge: a glycine-rich secreted salivary gland protein (Genbank:EU207085) and two unknowns (Genbank:EU211627) and (Genbank:EU209094). Group 2 included one transcript at 3 hours post challenge: a potassium: amino acid symporter (Genbank:EU210583). Group 3 included four transcripts at 6 hours post challenge: a mucin-like peritrophin (Genbank:EU206650), a glycine-rich secreted salivary gland protein (Genbank:EU207085), a potassium: amino acid symporter (Genbank:EU210583), and a serine protease (Genbank:EU205658). Finally, Group 4 included one transcript at 12 hours post challenge: a chitinase (Genbank:EU205713). All primer sequences used in microarray validation are presented in Table S1.

### Embedding, sectioning, and visualization of aldehyde-fixed mosquito thoraces

Thoraces from mosquitoes exposed to *B. pahangi*-infected and uninfected bloodmeals were separated from whole bodies at 6, 9, and 14 days after blood feeding by making transverse cuts along the cervical membrane and the first abdominal segment. The legs and thoracic ventral cuticle were partially removed by making a coronal cut along the mesosternum and tissues were fixed by immersion in 4% formaldehyde in 0.1 M phosphate buffer (pH 7.0). Thoraces then were prepared for light microscopy as described [21]. Briefly, aldehyde-fixed thoraces were dehydrated through a graded ethanol series, infiltrated in JB4-Plus resin (Electron Microscopy Sciences, Hatfield, PA), and anaerobically embedded in polyethylene molding trays. Coronal and transverse sections of 2.5 µm thickness were cut, stained with Gill's hematoxylin and Eosin Y, and mounted on glass slides using Poly-Mount (Polysciences Inc., Warrington, PA). Tissues were then imaged using differential interference contrast (DIC) optics on a Nikon Eclipse 90i compound microscope connected to a Nikon DS-Fi1 high-definition color CCD camera (Nikon Corp., Tokyo, Japan).

## Results

### *B. pahangi* development in *Ar. subalbatus*

The development of *B. pahangi* was observed microscopically in *Ar. subalbatus* at 1 h to 14 d (a total of 11 time points) post ingestion (PI) of a microfilaremic bloodmeal for the first biological replicate, and at 1 h and 14 d PI for the subsequent two biological replicates. The results are summarized in Table 1. Parasites were recovered from 70 of the 75 mosquitoes examined for an overall infection prevalence of 93.3%. Microfilariae were recovered from 1 h to 24 h PI, and constituted the majority of the total parasites through this time period. From 24 h to 3 d PI, almost all parasites had differentiated into intracellular L1s. L1s molted to L2s in the thoracic musculature by 5 d PI, and L2s were the only developmental stage identified between 5 and 6 d PI. The transformation from L2 to L3 occurred at 8–9 d PI. At 8 d PI, L2s and L3s were recovered from the thoracic musculature with only 2% of the total worms located in the head and proboscis. By 9 d PI all worms had molted to the L3 stage. At this time 43% of the L3s were observed in the thorax and 57% were located in the head and proboscis. By 13–14 d PI, all L3s were observed in the head and proboscis. The prevalence of L3s (for all three biological replicates on 14 d PI) was 87% (n = 15) and the mean intensity was 11.3±9.6 L3s.

**Table 1:** The development of *Brugia pahangi* in *Armigeres subalbatus* was recorded each time mosquitoes were collected for microarray analysis.

Time post feeding	Percentage of mosq. harboring worms (total dissected)	Developmental stage of *Brugia pahangi*				Total worms
		Microfilariae	L1	L2	L3	
1 hr	100% (15)	100% (152)				152
12 hr	100% (5)	42.2% (35)	57.8% (48)			83
24 hr	80% (5)	4.5% (1)	94.5% (21)			22
2 d	100% (5)	0% (0)	100% (38)			38
3 d	100% (5)	0% (0)	100% (28)			28
5 d	60% (5)			100% (7)		7
6 d	100% (5)			100% (55)		55
8 d	80% (5)			19.3% (11)	80.7% (46)	57
9 d	100% (5)			0% (0)	100% (49)	49
13 d	100% (5)				100% (61)	61
14 d	87% (15)				100% (150)	150

### Transcriptome changes in *B. pahangi*-infected vs. uninfected mosquitoes

**Figure 1:**
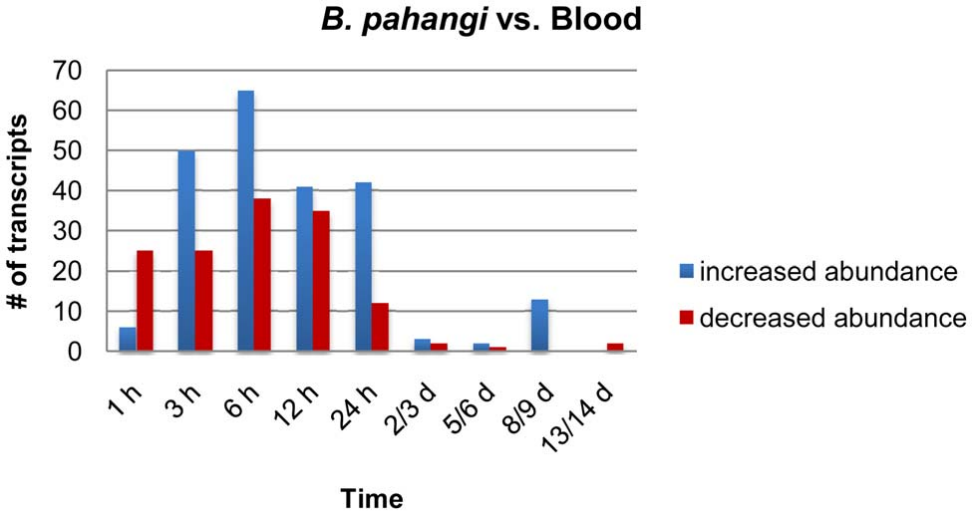
Transcriptional changes in *Armigeres subalbatus* following a *Brugia pahangi-*infected bloodmeal. The bar graph represents the number of significantly changed transcripts over the course of experimentation. Volcano plots were used to create working gene lists to identify differentially expressed transcripts at each time point. At 1, 3, 6, 12, 24 h post infection and 2–3, 5–6, 8–9, and 13–14 d post challenge there were 29, 75, 103, 76, 54, 5, 3, 13, and 2 detectable transcripts, respectively, with significantly different transcriptional behavior (increased or decreased transcript abundance at a 95% confidence interval over two-fold values) as a result of parasite development. The sample groups chosen are defined by the time post ingestion of the bloodmeal and represent significantly different stages of parasite development. The bar graph represents groups of mosquitoes that included those exposed to an infective bloodmeal containing *B. pahangi* mf (∼15–60 mf/20 µl blood) and those exposed to a bloodmeal without parasites.

Direct comparisons were made at each time point within DNA microarrays hybridized with probes made from RNA of whole female *Ar. subalbatus* exposed to either a *B. pahangi-*infected or uninfected bloodmeal. Volcano plots were used to create working gene lists to identify differentially expressed genes at each time point. At 1, 3, 6, 12, 24 h PI and 2–3, 5–6, 8–9, and 13–14 d PI there were 31, 75, 103, 76, 54, 5, 3, 13, and 2 detectable transcripts of the 6,143 features on the DNA microarray, respectively, with significantly different transcriptional patterns (increased or decreased transcript abundance at a 95% confidence interval over two-fold values) as a direct or indirect result of parasite development (Figure 1). Between each time point there was very little overlap (i.e., less than 27 transcripts shared between any two timepoints) in the transcripts that showed significantly different transcriptional patterns (Table 2).

**Table 2:** Few *Armigeres subalbatus* genes are differentially transcribed in multiple stages of the infection response to *Brugia pahangi*.

	1 h	3 h	6 h	12 h	24 h	2/3 d	5/6 d	8/9 d	13/14 d
**1 h**	33	4	5	5	4	0	1	1	1
**3 h**	θ	75	27	14	12	0	0	0	0
**6 h**	θ	θ	103	16	14	0	1	1	1
**12 h**	θ	θ	θ	76	12	0	0	2	0
**24 h**	θ	θ	θ	θ	54	0	1	2	1
**2/3 d**	θ	θ	θ	θ	θ	5	0	0	0
**5/6 d**	θ	θ	θ	θ	θ	θ	3	1	1
**8/9 d**	θ	θ	θ	θ	θ	θ	θ	13	1
**13/14 d**	θ	θ	θ	θ	θ	θ	θ	θ	2

Between each time point there was very little overlap in the transcripts that showed significantly different transcriptional behavior. This incongruity between the time points suggests that there is a great deal of informative and continual change in the transcriptome of *Armigeres subalbatus* in response to infection with *Brugia pahangi*.

**Figure 2:**
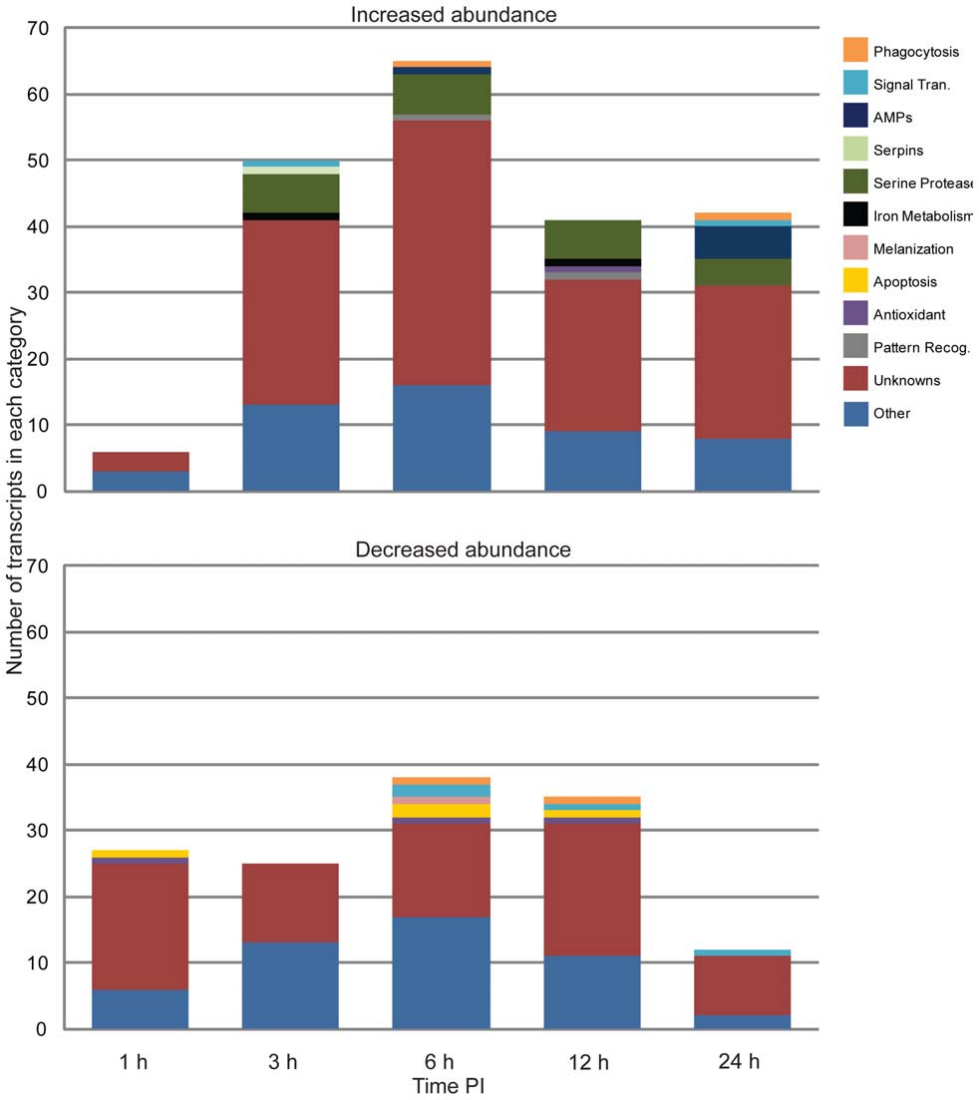
Functional composition of transcripts significantly affected by parasite infection. The categories are based on abundant and immunity-related EST clusters observed from *Armigeres subalbatus* cDNA libraries. Transcripts with a detectable increase in abundance (top) and with a detectable decrease in abundance (bottom) 1, 3, 6, 12, and 24 hours after exposure to a *Brugia pahangi* infective blood meal.

The vast majority of changes in the mosquito transcriptome occurred within the first 24 h of infection, when mf were penetrating through the midgut and invading thoracic muscle cells. In contrast, relatively minor changes in the mosquito's transcriptome were noted at later times during parasite development. This is somewhat surprising, considering that at 5–6 d PI parasites were actively feeding on mosquito tissue, and by 13–14 d PI parasites had grown considerably in size and migrated to the mosquito's head and proboscis. Of the transcripts that showed significantly different transcriptional patterns over this experiment, only 10% had putative immune functions (Table 3). In addition, of the 364 transcripts that showed significantly different transcriptional patterns over the time course of the *B. pahangi* vs. blood experiment, 193 (53%) of those transcripts were unknowns or conserved unknowns, i.e., they have no previously described function (Figure 2). This suggests that the function of many factors involved in the infection response to developing filarial worms (i.e., the factors involved in helping the mosquito maintain homeostasis during a persistent infection) are not known. Table S2 provides a full representation of all transcripts showing a detectable increase or decrease in abundance at all time points.

**Table 3:** Analysis of *Armigeres subalbatus* immunity genes transcribed by *Brugia pahangi-*infected mosquitoes.

Genbank ID	Description	Category	Relative Transcript Abundance^a^				
			1 h	3 h	6 h	12 h	24 h
EU211663	peptidoglycan recognition protein LE	PAT RECG			2.51		
EU205423	C-type lectin	PAT RECG				2.82	
EU206838	phenylalanine hydroxylase	MELN			−2.15		
EU209622	serine protease	MELN		2.65	3.51	6.17	
EU205154	serine protease	MELN		4.32	4.44	3.89	2.41
EU208339	serine protease	PROT		3.62	3.40		2.09
EU208787	serine protease	PROT		2.47			
EU206217	serine protease	PROT			2.05		
EU205815	serine protease	PROT				3.12	2.29
EU207063	serine protease	PROT				2.84	
EU205658	serine protease	PROT		4.39	5.91	3.21	2.06
EU208659	cecropin C	AMP			3.43		3.66
EU210693	cecropin A	AMP					4.61
EU205505	lysozyme	IMMUN					3.13
EU21110	holotricin 3	IMMUN					2.57
EU205824	defensin	AMP					2.24
EU205803	oxidoreductase	ANTIOX				2.17	
EU208063	thioredoxin	ANTIOX	−8.70				
EU211799	oxidoreductase	ANTIOX			−2.62		
EU206910	thioredoxin peroxidase	ANTIOX				−11.45	
EU206583	transferrin 1	IRON		2.58		2.28	
EU212056	serpin	PHAG		2.19			
EU206038	serine protease	PHAG		4.55	3.67		
EU209050	monophenol monoxygenase activator	PHAG			3.65		
EU206136	G protein-coupled receptor kinase	PHAG			−7.09		
EU207422	protein phosphatase regulator	PHAG				−29.24	
EU205197	gelsolin	PHAG					2.06
EU212174	serine protease	SIGNL				2.03	
EU205809	protein kinase C inhibitor	SIGNL		2.14			
EU205109	receptor signaling protein	SIGNL			−2.15		
EU205867	juvenile hormone epoxide hydrolase	SIGNL				−2.23	
EU205966	transmembrane protein serine/threonine kinase	SIGNL					2.27
EU209623	protein kinase	SIGNL					−2.56
EU210294	calcium-independent phospholipase	APOPT	−9.17				
EU205045	protein transporter	APOPT			−2.06		
EU210658	protein transporter	APOPT			−2.50		
EU206601	cathepsin	APOPT				−2.11	
EU206650	mucin-like peritrophin	IMMUN		2.06	2.58		

a Fold change (p<0.05), by time post infection.

PAT RECG =  Pattern Recognition, MELN =  Melanization, PROT =  Proteolysis, AMP =  Antimicrobial peptide, IMMUN =  Immune Response, ANTIOX =  Antioxidant, IRON =  Iron Metabolism, PHAG =  Phagocytosis, SIGNL =  Signal Transduction, APOPT =  Apoptosis.

### Transcriptome changes in *B. pahangi*- vs. *B. malayi*-exposed mosquitoes

**Figure 3:**
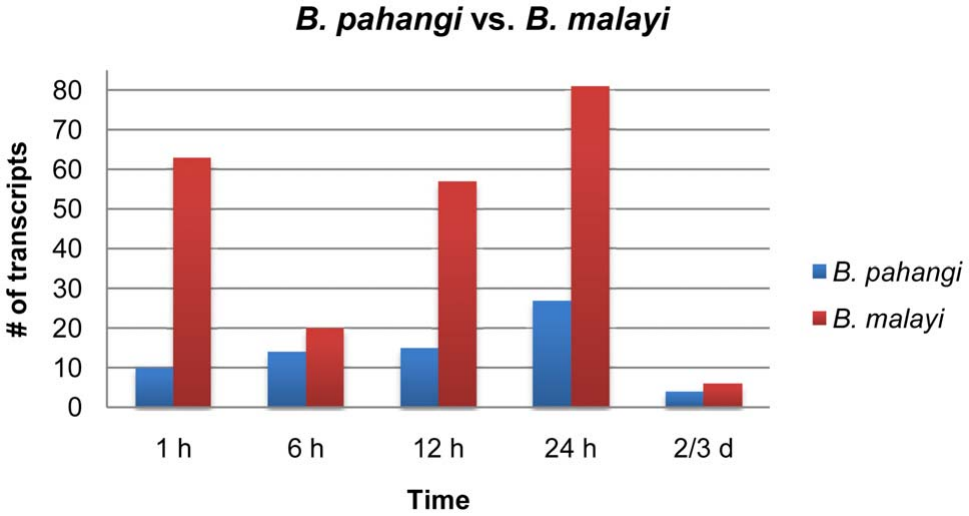
Transcriptional changes in *Armigeres subalbatus* when comparing a *Brugia pahangi- vs*. a *Brugia malayi-*infected bloodmeal. The bar graph represents the number of genes with a significant fold difference between *B. pahangi-* or *B. malayi-*exposed mosquitoes. Volcano plots were used to create working gene lists to identify transcripts associated with *B. pahangi* development (left) vs. *B. malayi* resistance (right). At 1, 6, 12, 24 h post infection and 2–3 d post challenge there were 10, 14, 15, 27, and 4 detectable transcripts, respectively, more associated with *B. pahangi* infection. Following the same time course, there were 63, 20, 57, 81, and 6 detectable transcripts, respectively, more associated with *B. malayi* resistance. The bar graph represents groups of mosquitoes that included those exposed to blood containing *B. pahangi* mf and those exposed to blood containing *B. malayi* mf. Significant increases or decreases in transcript abundance cannot be delineated.

Direct comparisons were made at each time point within DNA microarrays hybridized with probes made from RNA of whole female *Ar. subalbatus* exposed to either a *B. pahangi-* or *B. malayi-*infective bloodmeal. Volcano plots were used to create working gene lists to identify genes with a significant fold difference to begin to better understand the transcriptional profiles associated with exposure to each species of parasite. At 1, 6, 12, 24 h PI and 2–3 d post challenge there were 10, 14, 15, 27, and 4 detectable transcripts, respectively, more associated with *B. pahangi* infection relative to *B. malayi* resistance. Following the same time course, there were 63, 20, 57, 81, and 6 detectable transcripts, respectively, more associated with *B. malayi* resistance relative to *B. pahangi* infection (Figure 3).

**Figure 4:**
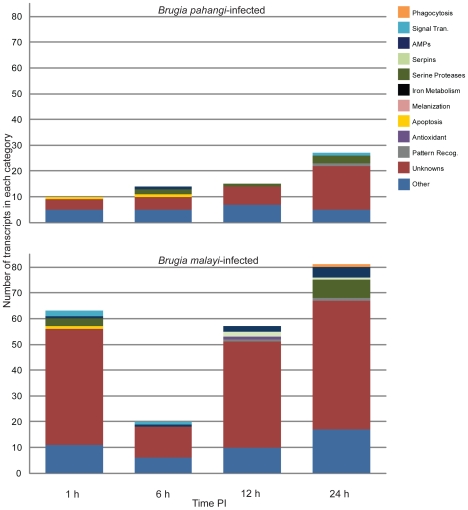
Functional composition of transcripts associated with *Brugia pahangi* infection or *Brugia malayi* resistance. The categories are based on abundant and immunity-related EST clusters observed from *Armigeres subalbatus* cDNA libraries. Transcripts associated with *B. pahangi* infection (top). Transcripts associated with *B. malayi* resistance (bottom).

The majority of changes that occurred in the mosquito transcriptome were associated with *B. malayi* resistance (76%); however, there may be some bias in these data because there was no *B. pahangi*-exposed mosquito cDNA included in the EST libraries used to create our *Armigeres* microarray [17]. Of the transcripts that showed significantly different transcriptional patterns as a result of *B. pahangi* infection, only 14% had putative immune functions (Table 4) and 49% were unknowns or conserved unknowns. Of the transcripts that showed significantly different transcriptional patterns as a result of *B. malayi* resistance, only 12% had putative immune functions (Table 4) and 67% were unknowns or conserved unknowns. These results suggest that although the biosynthetic pathway of melanization is well understood, many factors involved in the anti-filarial worm immune response are not known. Filarial worm susceptibility in *Ar. subalbatus* is much more complicated than merely an absence of the melanization immune response (Figure 4); likewise, filarial worm resistance in *Ar. subalbatus* is much more complicated than the presence of melanization and likely includes factors involved in recovery and maintenance of homeostasis. For example, there were few transcripts involved in the metabolism of reactive intermediates that showed significantly different transcriptional patterns as a result of *B. pahangi* infection vs. uninfected blood (Table 3). This is in contrast to what was observed in *Ar. subalbatus* in response to *B. malayi* [11], and probably has to do with the fact that mosquitoes infected with *B. pahangi* do not have to protect themselves from the damaging effects of the oxidative stressors produced during melanization reactions [22]. Table S3 provides a full representation of all transcripts showing a detectable difference in abundance between *B. pahangi-* and *B. malayi-*infected mosquitoes at all time points.

**Table 4:** Analysis of *Armigeres subalbatus* immunity related genes transcribed by either *Brugia pahangi-* or *Brugia malayi-*exposed mosquitoes.

Genbank ID	Description	Category	Relative Transcript Abundance^a^			
			1 h	6 h	12 h	24 h
***B. pahangi***						
EU206366	G-protein coupled receptor	APOPT	2.10			
EU208958	cathepsin	APOPT		2.97		5.80
EU205277	defensin A	AMP		2.93		
EU206217	serine protease	PROT		2.23	2.03	
EU206528	serine protease	PROT				2.84
EU208879	serine carboxyprotease	PROT		2.18		2.43
EU207696	serine protease	IMMUN				2.22
EU206649	calreticulin	PAT RECG				2.13
EU205878	*Ae. aeygpti* putative protein G12	SIGNL				2.07
***B. malayi***						
EU212480	serine protease	PROT	2.20			
EU212003	serine protease	PROT	2.75			
EU208214	serine protease	PROT	2.03			
EU208787	serine protease	PROT				2.28
EU207063	serine protease	PROT				2.29
EU205658	serine protease	PROT				2.75
EU205450	serine protease	PROT				3.42
EU205154	serine protease	MELN				2.95
EU209622	serine carboxyprotease	PROT				3.95
EU206047	serpin 27A	MELN				2.91
EU206540	mannose-binding lectin assoc. ser. prot.	SIGNL				2.35
EU209082	salivary secreted serpin	PROT			2.50	
EU211889	immune reactive protease inhibitor	IMMUN			6.58	
EU208239	toll-7	SIGNL	2.11			
EU211110	holotrocin 3	IMMUN				2.98
EU211628	diptericin B	IMMUN	3.16	2.09		
EU208970	CD36-protein	SIGNL	2.44			
EU208273	scavenger receptor	SIGNL		7.81		
EU207965	defensin A	AMP				2.47
EU205465	defensin C2	AMP			3.64	
EU206070	cecropin	AMP				4.93
EU205409	cecropin B1	AMP			2.84	3.24
EU206422	gambicin	AMP				2.16
EU206257	c-type lectin	PAT RECG				2.29
EU208542	c-type lectin	PAT RECG			2.56	2.53
EU208009	glutathione transferase	ANTIOX			2.10	
EU209050	monophenol monooxygenase activator	PHAG				2.18
EU206650	mucin-like peritrophin	IMMUN				2.39

a Fold difference between *B. pahangi* and *B. malayi* (p<0.05), by time post infection.

PAT RECG =  Pattern Recognition, MELN =  Melanization, PROT =  Proteolysis, AMP =  Antimicrobial peptide, IMMUN =  Immune Response, ANTIOX =  Antioxidant, IRON =  Iron Metabolism, PHAG =  Phagocytosis, SIGNL =  Signal Transduction, APOPT =  Apoptosis.

### Microarray validation

Microarray data were confirmed using both *in silico* analyses of known transcriptional information in the literature and laboratory-based analyses via qPCR [18], [23], [24]. The transcriptional activity of a number of different response genes, induced by bacteria or filarial worms, has been characterized previously in several mosquito species. Based on this information, and the fact that the RNA used to screen the DNA microarrays in these studies result from the exposure to filarial worms, it was expected that a number of parasite responsive genes on the DNA microarrays would show significant transcriptional patterns [11], [25]–[30]. Comparisons made between our data and parasite responsive genes in the literature corroborated our findings and provided validation of our DNA microarray results. In addition, a number of “house-keeping” genes (ex. Ribosomal genes, actin, cytochrome C oxidase, etc.) included on the DNA microarray showed no detectable change in transcript abundance throughout experimentation (data not shown), thereby providing further validation of the expression patterns detected.

In conjunction with *in silico* validation of DNA microarray results, qPCR provided independent, experimental verification of transcript abundance from the same total RNA used in the initial DNA microarray experiment. Because the corroboration of all microarray data was impractical, a subset of seven genes was chosen at random from our lists of significant genes for confirmatory studies. Transcriptional activity of three selected genes was verified at 1 h, one selected gene at 3 h, four selected genes at 6 h, and one selected gene at 12 h post challenge, and eight of the nine conditions tested corroborated with transcriptional patterns detected on the DNA microarray (Text S1). The elimination of one gene from our dataset may reflect the differential sensitivities of the techniques used or sample variation. This still validated that the DNA microarray was working as expected, showing all three conditions (increase, decrease, and no detectable change in transcript abundance). These results, in combination with *in silico* and laboratory-based validation, provided confidence that the transcriptional profiles are an accurate depiction of the biological phenomena under study.

### Histology

**Figure 5:**
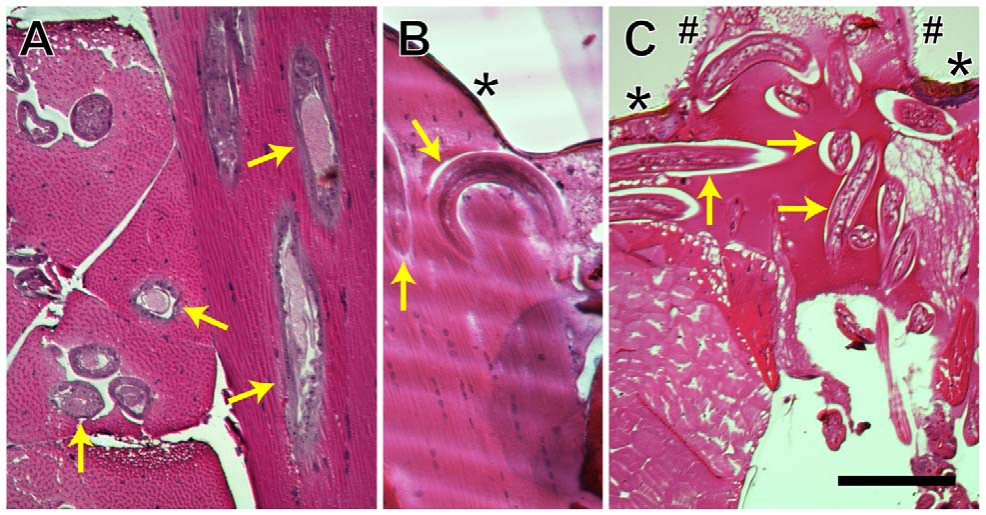
Development of *Brugia pahangi* in the mosquito thorax. At 6 days post-infection parasites (e.g., arrows) are developing in the thoracic musculature and are oriented parallel to the myofibers (**A**). At 9 days post-infection parasites exit the myofibers (**B**) and migrate to the cervix and head region (**C**; 14 days post-injection). *, thoracic cuticle; #, cervical cuticle; scale bar, 100 µm.

Histological analyses of thoraces from infected mosquitoes confirmed the infection timeline observed using whole body dissections. At 6 d PI worms were developing in the thoracic musculature and, with rare exceptions, were oriented parallel to the muscle fibrils. At 9 d PI the worms were in the process of exiting the muscle fibers and migrating to the head, where they generally remain for the lifetime of the mosquito (Figure 5).

Because the microarray data showed remarkably few mosquito transcriptional changes associated with parasite maturation and migration in the hemocoel, we undertook a comparative histological analysis of the thoracic musculature from *B. pahangi*-infected and uninfected mosquitoes, specifically surveying for obvious morphological changes associated with worm infection. As indicators of tissue damage, particular attention was paid to signs of muscle fiber degradation, the structure of myofibrils that were in direct contact with the worms, the morphology of host muscle nuclei, and signs of tissue infiltration by fat body. To assess changes at different points of the filarial nematode's life cycle, these analyses were performed at 6, 9, and 14 d post exposure to infective and normal bloodmeals, because these represent times when the worms are actively feeding, migrating, and infective to the vertebrate host, respectively.

**Figure 6:**
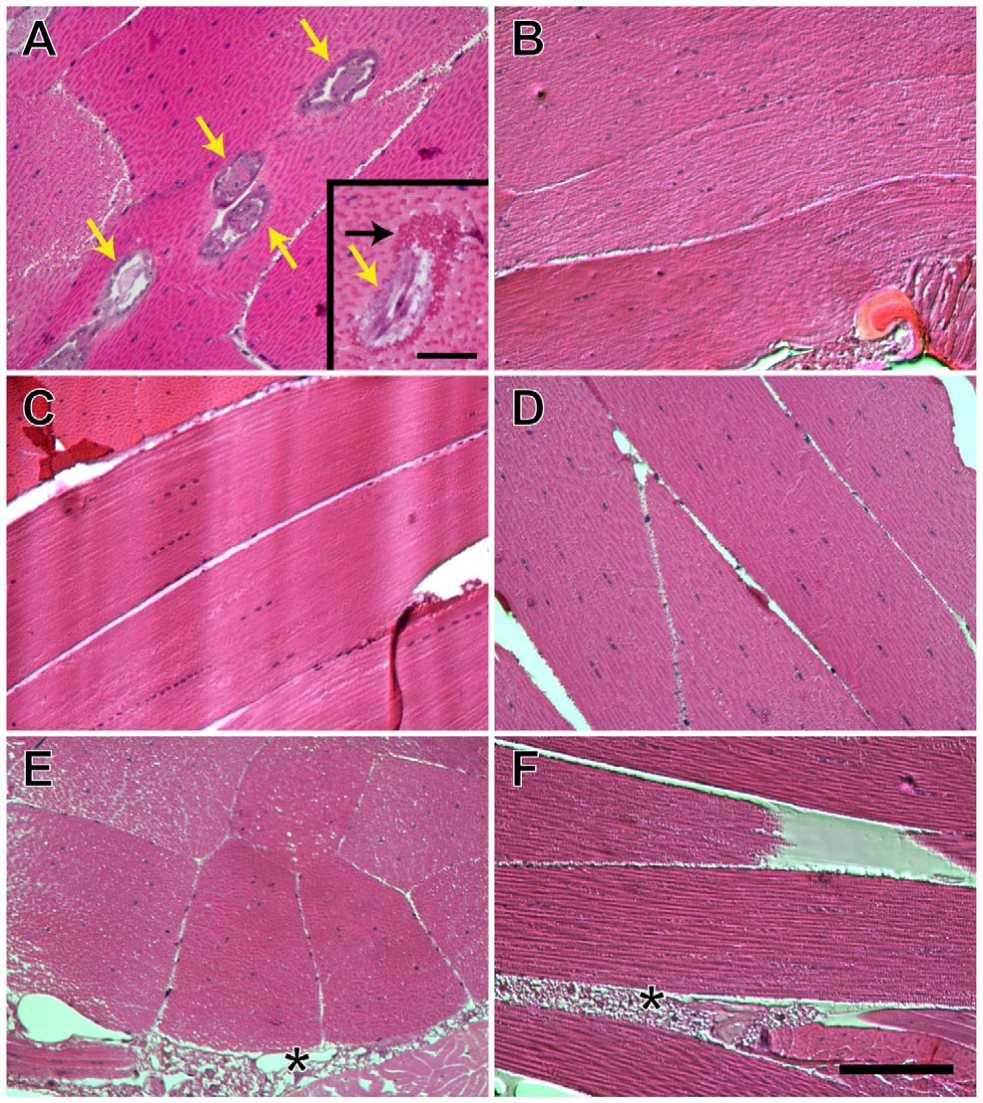
Comparative histological imaging of thoracic indirect flight muscles after *Brugia pahangi*-infected and uninfected bloodmeals. Aside from occasional host-derived tissue pooling between the worms and the myofibrils (**A, A inset**), no obvious pathology associated with infection was observed at 6 days (**A**, **B**), 9 days (**C**, **D**) or 14 days (**E**, **F**) after mosquitoes received *Brugia pahangi*-infected (**A**, **C**, **E**) and uninfected (**B**, **D**, **F**) bloodmeals. *B. pahangi*, yellow arrows; host tissue pooling, black arrow; *, fat body; A–F scale bar, 100 µm; A inset scale bar, 25 µm).

Overall, examination of hematoxylin and eosin-stained semi-thin sections did not reveal any obvious tissue damage associated with *B. pahangi* infection (Figure 6). At 6 d PI, greater than 10 worms were commonly observed in individual sections, indicating that infection intensities were high. Worms developed parallel to the myofibrils with little separation between the nematode cuticle and the mosquito myofibrils. As compared to non-infective bloodmeal controls, no obvious morphological change was observed in the infected muscle fibers, with the exception of what appeared to be occasional host-derived tissue pooling between the worm and the myofibrils. This tissue pooling, consisting of eosinophilic granules that we hypothesize are mitochondria, is likely the result of active feeding by the worm but surprisingly did not damage the integrity of the myofibers: sarcomere patterns were morphologically similar to those in uninfected mosquitoes. At 9 d PI, worms were observed migrating toward the head by laterally crossing the myofibers before exiting into the periphery of the thorax. Besides the physical breaks caused by worm migration, little additional damage was visually detected along the remaining portions of the invaded myofibers and the sarcomeres flanking these breaks displayed normal morphological patterns relative to adjacent myofibers and non-infected blood meal controls. By 14 d PI worms could only be detected near the head and were not intracellularly associated with the thoracic musculature. From the examination of the thoracic musculature we could not conclusively determine the precise location of earlier worm development, indicating that parasites do not leave behind obvious voids near their developmental areas and that much of the displacement caused by their presence is either repaired or filled by adjacent muscle fibers or fibrils.

At all timepoints assayed, both infected and uninfected mosquitoes contained small numbers of myofibers that were atrophied or in the process of degradation. However, at none of the timepoints assayed did we detect higher numbers of abnormal myofibers in infected as compared to uninfected mosquitoes, suggesting that this fiber atrophy is either related to normal biological turnover or an artifact of the tissue manipulation process (embedding, sectioning, etc.). Furthermore, fat body was observed near the periphery of the thorax and at times extended between muscle fibers. This also appeared to be a normal biological occurrence as a similar pattern of fat body distribution was observed in uninfected mosquitoes. Lastly, comparison of myofiber nuclear morphology in infected and uninfected mosquitoes revealed no obvious differences in nuclear size or chromatin condensation, further suggesting that infection does not result in significant muscle atrophy.

## Discussion

Interactions between mosquitoes and filarial worms can range from an almost benign symbiotic relationship between organisms, to a fatal competition resulting in the death of the host, or to a fatal competition resulting in the death of the parasite. In *Ar. subalbatus* both tolerance and resistance strategies can occur depending on the species of filarial worm infecting the mosquito, but high mosquito mortality, caused by ingestion of too many mf, can occur with either scenario. It is important to distinguish between tolerance and resistance because their relative importance will have substantial consequences for the ecology and evolution of host-parasite interactions [31]. Traditionally, most studies have examined the anti-filarial worm immune response in *Ar. subalbatus* (e.g., [11], [25], [29]), or explored innate immune responses to other pathogens in other mosquito species (e.g., [4], [32], [33]), but few have examined pathogen susceptibility in a natural mosquito vector (in this case, a laboratory strain). Studies like this one, aimed at understanding the molecular basis of parasite-host interactions in a compatible system, will help identify the components involved in host resistance vs. parasite tolerance [4].

It is important to note that resistance and tolerance can be mutually exclusive, interchangeable, or complementary components of a mixed strategy of defense [34], and a particular gene may be involved in both tolerance and resistance, depending on the pathogens involved [35]. Furthermore, tolerance may involve immunological mechanisms directed at damage or other harmful substances resulting from infection with the parasite, or may even reflect the parasite's ability to persistently evade the host's defenses to remain inside the host to achieve eventual transmission [36], which will complicate the elucidation of the factors determining filarial worm susceptibility in this species of mosquito. For example, a number of transcripts implicated in innate immunity showed significantly different transcriptional behavior as a result of *B. pahangi* infection vs. uninfected blood (e.g., serine proteases, pattern recognition molecules, etc) in the current study, and this may be an example of these transcripts being directed at damage or other harmful substances resulting from infection with the parasite.

Apoptosis also could explain the activity of a number of the immune responsive transcripts present in this study, because cell death in vertebrates has been shown to trigger both innate and adaptive immune responses [37], [38]. It also could explain the activity of those transcripts implicated in phagocytosis, because phagocytosis could be functioning to clean-up apoptotic cells [37]–[39] that were destroyed by mf penetrating the midgut. This is consistent with previous reports of apoptosis in the midgut of parasite and/or virus infected mosquitoes [40]–[46], and it has been shown previously that basal and apical plasma membranes are destroyed by mf penetrating the midgut, likely resulting in cell death [47].

A number of antimicrobial peptides (AMPs) showed significantly different transcriptional behavior in the current study as well. Anti-microbial peptides are small, immune-related molecules named for their *in vitro* activity against bacteria and are detectable in the fat body, hemocytes, midgut, and epithelial tissues of mosquitoes. Although considered a primary defense mechanism against bacteria in mosquitoes, AMP transcription has been associated with responses to *B. malayi* infection in *Armigeres* and *Aedes*, *Plasmodium* infection in *Anopheles*, and fungal infection in other mosquito species [11], [32], [48], [49]. Despite these associations, our knowledge of the molecular mechanisms and the true role of these peptides in mosquito innate immunity remain limited, and perhaps, the anti-microbial activity of AMPs might be an ancillary property. Recently, it has been shown that antimicrobial compounds function primarily to protect insects against bacteria that persist within the body, rather than to clear the infection. It has been proposed that AMPs may act as response elements helping insects deal with infection when pathogens in the hemolymph exceed the phagocytic or melanotic capacity of the hemocytes [50], [51]. The results of the current study are consistent with this hypothesis, i.e., the fact that AMP induction is evident as a result of *B. pahangi* infection in addition to *B. malayi* resistance seems to support the hypothesis that AMPs may play an alternative role, perhaps helping to maintain homeostasis of the hemolymph environment during a persistent infection or to clean up after a successful immune response, but are transcribed regardless of the type of pathogen present.

In the vertebrate host, filarial nematodes apply successful strategies to evade the host's immune response [52], [53]. Their strategy has evolved to be assimilation, defusing aggressive immune reactions, and inducing forms of immunological tolerance to permit their long-term survival [53]. In the *Armigeres*-*Brugia* system, *B. pahangi* may employ similar strategies; whereas, *B. malayi* is not successful in evading the mosquito's immune response. The specificity of resistance in this vector-parasite relationship warrants further exploration, and we postulate it to occur at the level of recognition, possibly involving well defined motifs (e.g., β 1,3-glucan [27]) recognized by receptors that could be the products of resistance genes. Immune defenses (e.g., melanization) would then be triggered only if recognition of the parasite occurs [4]. Therefore, the general hypothesis is that *Ar. subalbatus* recognizes a surface component(s) of *B. malayi* mf but not *B. pahangi* mf, i.e., there is a fundamental biologic difference in the surface components of the two filarial worm species. Consistent with this hypothesis is the fact that a number of transcripts associated with targeting/initiating an immune response changed as a result of *B. malayi* infection relative to *B. pahangi* infection: e.g., CD-36-protein (GenBank: EU208970), scavenger receptor (GenBank: EU208273), toll-like receptor 7 (GenBank: EU208239), 2 C-type lectins (GenBank: EU206257 and EU208542), and a mannose-binding lectin associated serine protease (GenBank: EU206540).

If vector-parasite specificity is not achieved at the recognition level, we postulate that specificity could be explained by direct or indirect interactions between the products of immune suppressive genes of the parasite and the products of host resistance genes [4], i.e., *B. pahangi* mf can actively suppress the anti-filarial worm response in *Ar. subalbatus* but *B. malayi* mf cannot. Consistent with this hypothesis is the fact that the majority of changes that occurred in the mosquito's transcriptome were associated with *B. malayi* resistance (76%) relative to *B. pahangi* susceptibility. In addition, indirect comparisons between *B. malayi* resistance [11] and *B. pahangi* susceptibility showed considerably more changes associated with resistance (761 vs. 346) over the course of 72 h.

Recently, we examined the infection response of *Ae. aegypti* to *B. malayi* at the transcriptional level [12]. In this experimental model system, essentially all ingested parasites successfully develop to L3s. This study revealed very few changes in the *Ae. aegypti* transcriptome until L2s were present, and the most profound transcriptional changes were observed in mosquitoes that harbored infective-stage parasites. In comparison, the vast majority of changes in the *Ar. subalbatus* transcriptome as a result of *B. pahangi* infection were observed between 1 and 24 hours PI when mf were penetrating the midgut and invading thoracic muscle cells, and very few transcriptional changes were observed in mosquitoes that harbored L2s or infective-stage parasites. It is important to note that the array platforms used to conduct these two studies were different (*Ar. subalbatus-B. pahangi*  =  EST-based DNA microarray; *Ae. aegypti-B. malayi*  =  whole genome DNA microarray) because there is no genome sequence available for *Ar. subalbatus*, and some of the differences observed between the two studies may be the result of the inherent differences in these two approaches. But the different transcriptional changes observed in these two mosquito species that support parasite development also can provide clues to the molecular mechanisms that determine compatibility in natural mosquito-filarial worm associations. By comparing the two infection responses we can begin to better understand the intricacies involved in susceptibility vs. refractoriness, i.e., by identifying transcripts that are shared between both compatible mosquito-parasite systems and from the refractory condition, we can begin to rule out their involvement in anti-filarial worm immunity. Such comparisons have been made previously between other mosquito genera (e.g., [54]), but this study is the first to make a comparison between the susceptible vs. refractory state in the same species of mosquito, and may provide a better representation of the genes required to deter filarial worm infection in an incompatible system. These results also illustrate the fact that not all mosquitoes respond the same way to filarial worm infection and not all filarial worm species will elicit the same response in a host. Host-parasite interactions represent coevolved adaptations of significant complexity, and these relationships depend on the relative capacities of the host and pathogen to adapt to and maintain this unique relationship.

Furthermore, earlier studies assessing *B. malayi* and *B. pahangi* infection of *Ae. aegypti* and *Mansonia uniformis* found that nematode migration and development causes minor and severe damage to the thoracic indirect flight muscles [55], [56]. Later, it was proposed that this damage was not pathogen specific because similar damage was observed in mosquitoes physically traumatized by intrathoracic insertion of a metal probe [57]. In contrast to those studies, the experiments we report here failed to detect significant pathology in the thoracic musculature of *Ar. subalbatus* infected with *B. pahangi*, with the exception of breaks in myofibers that appear to be the direct result of nematode exit from the indirect flight muscles. This is consistent with other studies assessing *B. pahangi* infection in mosquitoes, where infection of the natural vector *Aedes togoi* resulted in undetectable flight muscle damage (suggesting complete myofiber repair following migration to the head), whereas infection of the artificial vector *Ae. aegypti* resulted in severe degeneration of the flight muscles [58], [59]. The histological and transcriptomic data reported in this study further verify the need to work with natural mosquito-parasite systems, because it is evident that extrapolating what is learned from a laboratory model of a parasite-vector relationship to the natural model can be problematic.

Aside from myofiber breaks, the primary difference between the thoracic musculature from infected versus normal bloodfed *Ar. subalbatus* was the accumulation of eosinophilic granules between the nematode cuticle and the host myofibers. We presume that these granules are host mitochondria, and if so, these data would be in accord with electron microscopic data showing similar accumulations following *B. pahangi* infection of *Ae. aegypti* [60]. In that study, pooled mitochondria showed no evidence of damage and it is not clear if any deleterious effect is associated with their accumulation. However, because similar structures were observed in the nematode gut [60], it is possible that filarial nematodes subsist by ingesting mitochondria. Our data and that of others [60] have provided no evidence that filarial nematodes ingest the contractile components of the thoracic musculature.

Although our data did not reveal any obvious pathological consequence to infection, pathology may still exist. The techniques used in this study only allowed for the examination of structural damage at the light microscopic level and did not molecularly assess cell death, or explore pathology at the ultrastructural level. Significant mortality has been associated with filarial nematode invasion of and exit from the indirect flight muscles of *Ar. subalbatus* (Aliota et al., unpublished; and [61]) and it is possible that this mortality is a consequence of damage that impairs flight, resulting in decreased feeding and other essential biological processes.

Finally, it is important to consider that host and parasite genotypes share control of epidemiological parameters of their relationship. Most models of the evolutionary processes in host–parasite systems assume that the evolution of attack or defense strategies is governed by the balance of their evolutionary costs and benefits from the point of view of either the parasite or the host and, thus, hold the other partner constant. In other words, they consider that the traits of the relationship are determined by the genotype either of the host or of the parasite, but not an interaction between the two [62]. Future studies that explore the Interactome- the whole set of molecular interactions of both organisms in a symbiotic relationship- of a host-pathogen system should be extremely valuable in determining the evolutionary basis for tolerance vs. resistance and help to elucidate the underlying components of vector competence.

## Supporting Information

Table S1 PCR primer sequences.
(0.05 MB DOC)

Table S2 Analysis of all *Armigeres subalbatus* genes transcribed by *Brugia pahangi*-infected mosquitoes. A tabular representation of all transcripts showing a detectable increase or decrease in abundance at all time points. The table lists the ASAPID number, associated product, GO designation, and relative transcript abundance. All time points are represented.
(0.08 MB XLS)

Table S3 Analysis of all *Armigeres subalbatus* genes transcribed by either *Brugia pahangi*- or *Brugia malayi*-infected mosquitoes. A tabular representation of all transcripts showing detectable differences in abundance at all time points. The table lists the ASAPID number, associated product, GO designation, and relative transcript abundance expressed as the fold difference between mosquitoes infected with either parasite. All time points are represented.
(0.06 MB XLS)

Text S1 Correlation of log2 ratios from microarray expression data with log2 qPCR expression values. Validation of microarray data with qPCR. The expression values (log2 ratios) for seven genes in four separate time points are plotted against the RT-qPCR expression values. The Pearson's correlation coefficient of 0.702 and the goodness of the fit (R^2^ = 0.500) indicates a high degree of correlation.
(0.07 MB DOC)
