# Supplementary material for: Mosquito Transcriptome Profiles and Filarial Worm Susceptibility in Armigeres subalbatus
Source: PLoS Negl Trop Dis. 2010 Apr 20;4(4):e666. doi: 10.1371/journal.pntd.0000666 (PMC2857672; doi:10.1371/journal.pntd.0000666)
Supplement: Table S1 — PCR primer sequences. (0.05 MB DOC) [file pntd.0000666.s001.doc]

**Table S1**.- Primer sequences used in the validation of microarray data.

|  |  |  |  |  |
| --- | --- | --- | --- | --- |
| **GenBank ID** | **Primer sequence** |  |  |  |
| EU207085-F | 5'-TTCTCAAGGAGTCGGAATGG -3' |  |  |  |
| EU207085-R | 5'-TCAATGCGTATCGCTTTCAC -3' |  |  |  |
|  |  |  |  |  |
| EU2116227-F | 5'-GCGTGTGAGAACAGTGGAAG -3' |  |  |  |
| EU2116227-R | 5’-ATCCTTTTGACCGTGCTTCA -3’ |  |  |  |
|  |  |  |  |  |
| EU209094-F | 5'-ATTTTCCCGGATAGCCAAAT -3' |  |  |  |
| EU209094-R | 5'-TCGACACCGAAATTCTTTCC -3' |  |  |  |
|  |  |  |  |  |
| EU210583-F | 5'-CCAGCTCGAAAACGATTGAT -3' |  |  |  |
| EU210583-R | 5'-TTCCTCCTGGTAGCGAAGTC -3' |  |  |  |
|  |  |  |  |  |
| EU206650-F | 5'-ACAGCTCCACCGGTAACAAC -3' |  |  |  |
| EU206650-R | 5'-TTTCATTGCGCAGTTGACTC -3' |  |  |  |
|  |  |  |  |  |
| EU205658-F | 5'-GCTATCCTGCAGTCACACGA -3' | |  |  |
| EU205658-R | 5'-AGTGGGCAGCAGTGAGAACT -3' | |  |  |
|  |  |  |  |  |
| EU205713-F | 5'-CGGAAACAAAGCTGCCTATG -3' |  |  |  |
| EU205713-R | 5'-CTCGCAAAATGGGGAACTTA -3' |  |  |  |
|  |  |  |  |  |
|  |  | |  |  |
|  |  | |  |  |
|  |  |  |  |  |
|  |  |  |  |  |
|  |  |  |  |  |
