# Supplementary material for: Mosquito Transcriptome Profiles and Filarial Worm Susceptibility in Armigeres subalbatus
Source: PLoS Negl Trop Dis. 2010 Apr 20;4(4):e666. doi: 10.1371/journal.pntd.0000666 (PMC2857672; doi:10.1371/journal.pntd.0000666)
Supplement: Text S1 — Correlation of log2 ratios from microarray expression data with log2 qPCR expression values. Validation of microarray data with qPCR. The expression values (log2 ratios) for seven genes in four separate time points are plotted against the RT-qPCR expression values. The Pearson's correlation coefficient of 0.702 and the goodness of the fit (R2 = 0.500) indicates a high degree of correlation. (0.07 MB DOC) [file pntd.0000666.s004.doc]

**Text S1**

**
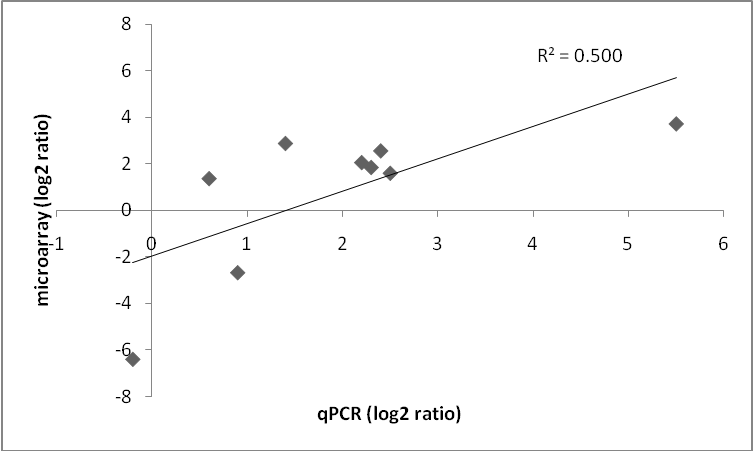
**

Validation of microarray data with qPCR. The expression values (log_2_ ratios) for seven genes in four separate time points are plotted against the RT-qPCR expression values. The Pearson’s correlation coefficient of 0.702 and the goodness of the fit (R2 = 0.500) indicates a high degree of correlation.

**Correlation of log_2_ ratios from microarray expression data with log_2_ qPCR expression values.**

| Group | GenBank ID |  | Log_2_ ratio | |
| --- | --- | --- | --- | --- |
|  |  | ASAP ID | qPCR | Microarray |
| Group 1 | EU207085 | ACN-0181041 | 1.40 | 2.88 |
|  | EU211627 | ACN-0182237 | 0.90 | -2.67 |
|  | EU209094 | ACN-0180347 | -0.20 | -6.39 |
|  |  |  |  |  |
| Group 2 | EU210583 | ACN-0186842 | 2.50 | 1.60 |
|  |  |  |  |  |
| Group 3 | EU206650 | ACN-0181224 | 0.60 | 1.37 |
|  | EU207085 | ACN-0181041 | 5.50 | 3.72 |
|  | EU210583 | ACN-0186842 | 2.30 | 1.85 |
|  | EU205658 | ACN-0181325 | 2.40 | 2.56 |
|  |  |  |  |  |
| Group 4 | EU205713 | ACN-0186097 | 2.20 | 2.06 |
|  |  |  |  |  |
